# ALK-positive advanced non-small cell lung cancer patients with poor performance status: Outcomes in a real-world scenario

**DOI:** 10.3332/ecancer.2022.1407

**Published:** 2022-06-07

**Authors:** Ajaykumar Singh, Akhil Kapoor, Vanita Noronha, Vijay Patil, Nandini Menon, Abhishek Mahajan, Amit Janu, Nilendru Purandare, Rajiv Kaushal, Kumar Prabhash

**Affiliations:** 1 Medical Oncology, Tata Memorial Centre, Mumbai 400012, India; 2 Medical Oncology, Homi Bhabha Cancer Hospital, Varanasi 221001, Uttar Pradesh, India; 3 Radiology Department, Medical Oncology, Tata Memorial Centre, Mumbai 400012, India; 4 Nuclear Medicine, Medical Oncology Tata Memorial Centre, Mumbai 400012, India; 5 Pathology, Medical Oncology Tata Memorial Centre, Mumbai 400012, India

**Keywords:** NSCLC – non small cell lung cancer, ECOG – Eastern Cooperative Oncology Group, OS – overall survival, PFS – progression-free survival;, IHC – immunohistochemistry, FISH – fluorescence in situ hybridisation

## Abstract

**Background:**

Anaplastic lymphoma kinase (ALK) inhibitors have shown significant efficacy in ﻿ALK -rearranged non-small cell lung cancer (NSCLC) patients with good performance status (PS) in multiple randomised studies. However, there is limited data on patients with poor performance status.

**Patients and methods:**

We carried out a retrospective analysis of prospectively collected data of patients with ALK-rearranged NSCLC and Eastern Cooperative Oncology Group (ECOG) PS of 2–4 treated at a single academic cancer centre from January 2013 to November 2018. The outcomes, progression-free survival (PFS) and overall survival (OS) were calculated from the date of diagnosis. SPSS version 20 was used for all statistical calculations.

**Results:**

Out of the total 441 ALK-positive patients, 97 (21.9%) had ECOG PS 2–4 (poor PS). The median PFS was 9.3 months (95% CI = 6.6–12.0) as compared to 14.9 months (95% CI = 13.4–16.4) for patients with a PS of 0–1 (HR = 1.38, 95% CI = 1.04–1.84, *p* = 0.027). The corresponding median OS were 17.9 months (95% CI = 12.8–23.1) and 33.5 months (95% CI = 28.6–38.4), respectively (HR = 1.89, 95% CI = 1.36–2.62, *p* < 0.001). Among poor PS patients, a subgroup of patients with PS 2 had median OS of 20.6 months (95% CI = 10.8–47.3) as compared to 8.6 months for PS 3–4 (95% CI = 7.8–27.8) (HR = 1.79, 95% CI = 1.01–3.20, *p* = 0.047). The patients treated with upfront ALK inhibitors had better survival as opposed to those treated with chemotherapy. On multivariate analysis, PS 3–4, smoking, stage 4 and not using ALK inhibitors as first-line therapy were associated significantly with poor outcomes.

**Conclusion:**

The ALK-rearranged NSCLC patients with poor PS derived significant benefits with ALK inhibitors. The outcomes were significantly poorer as compared to patients with PS 0–1; the subgroup of patients with PS 2 had better outcomes as compared to patients with PS 3–4.

## Introduction

Poor performance status (PS) is one of the most important prognostic factors in non-small cell lung cancer (NSCLC). Very few studies have included NSCLC patients with poor PS in chemotherapy or targeted therapy trials. In a study by Rogerio *et al* [1], NSCLC had the highest prevalence of poor PS. It showed that poor PS prevalence was 34% when estimated by providers and 48% when estimated by the patients themselves.

Anaplastic lymphoma kinase (ALK) rearrangements are the most common targetable oncogenic driver mutations found in never smokers who are wild-type for epidermal growth factor receptor mutation [2]. In the past, before the availability of ALK inhibitors, most patients were treated with conventional chemotherapy with platin-based regimens. The chemotherapy studies included very few patients with PS 2, as chemotherapy is known to have significantly higher toxicities as compared to various ALK inhibitors, especially in the PS 2 subgroup [3–5].

In today’s era, multiple ALK inhibitors, viz. crizotinib, ceritinib, alectinib, brigatinib and lorlatinib, have been approved. They have shown significant improvement in overall response rates (ORR), progression-free survival (PFS) and overall survival (OS) as compared to chemotherapy as the first-line or subsequent lines of therapy with less toxicity as compared to conventional chemotherapy [6–9] In most of these trials, only patients with good PS have been included. Very few studies included a limited number of patients with PS 2. There are very few prospective studies for the treatment outcomes in patients with poor PS, especially PS 3 or 4. Thus, it becomes difficult to have an evidence-based approach for these patients. In these situations, the real-world retrospective data can come to the rescue and help in the decision-making in these situations. Considering this, we carried out a retrospective analysis of the prospectively collected patient database of ALK-positive advanced lung cancer with poor PS.

## Patients and methods

This study is a retrospective analysis of prospectively collected data of patients treated at a single academic cancer centre from January 2013 to November 2018. Out of all the patients from this database, patients with ALK rearrangement-positive advanced lung cancer having poor PS, defined as Eastern Cooperative Oncology Group (ECOG) PS 2–4, who were registered between January 2013 and September 2018, were included in this study. The patients who were not found to harbour ALK rearrangement were excluded. No sample size calculation was carried out as all the patients who were registered in the lung audit during the specified time period were evaluated for the study. The data included baseline demographics, comorbidities, habits, ALK testing method and disease sites, in addition to the dates of diagnosis, the start of first-line therapy, progression, the start of a subsequent line of treatment, last follow-up and death (if occurred). The best response to the treatment and sites of progression was also recorded. The primary outcome of this study was the estimation of PFS, while the secondary outcomes included OS and toxicities. The details of pre-treatment evaluation and ALK testing have been mentioned in detail in our previous publication [10].

Response assessment was carried out every 8–12 weeks of therapy or earlier as per the treating physician’s discretion if clinically indicated and measured as per RECIST version 1.1 criteria. PFS was defined as the duration between the date of starting treatment and the date of disease progression or change in therapy or death, if it occurred before disease progression. OS was defined as time duration from the date of diagnosis of advanced-stage disease to date of death or last follow-up date. The data were analysed using SPSS for Windows version 20 (Armonk, NY, USA). Descriptive statistics were used to analyse demography data. The Kaplan–Meier method was used to calculate PFS and OS, and the comparisons of survival were made using the log-rank test. The Wilcoxon signed-rank test was carried out to check for the significance of change in ECOG PS post-first-line therapy.

## Results

### Baseline details

Out of 441 patients, 97 (22%) patients had poor PS at presentation. The median age of the patients was 53 years (range = 24–74), with 29 (29.9%) being >60 years at presentation; 43 (44.3%) were female and 37 (38.1%) had comorbidities. Smoking habit was present in 12 (12.4%), while 18 (18.6%) were tobacco chewers. Brain metastasis was present at baseline in 25 (25.8%) patients (Table 1). ALK testing was conducted with the immunohistochemistry (IHC) method in 80 (82.4%) patients, by FISH method in 13 (13.4%) patients and by both methods in 4 (4.2%) patients.

### Treatment details

**Figure 1.**
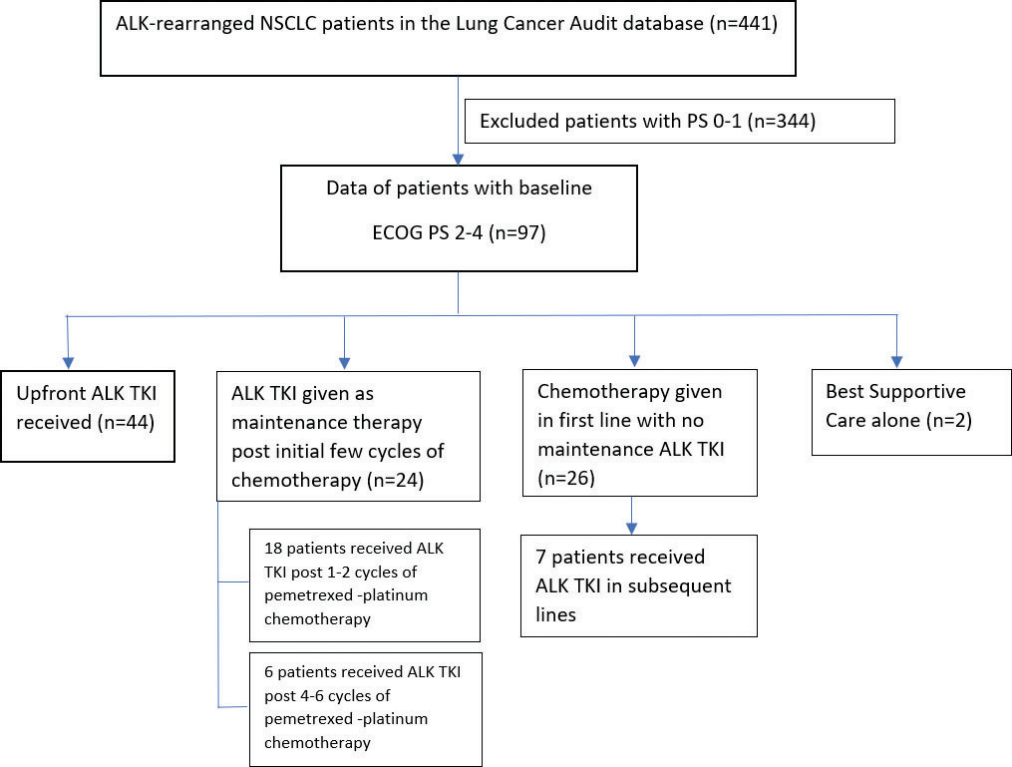
Consort diagram of the study.

Figure 1 shows the consort diagram of the study; 43 (44%) patients received upfront TKI (41 crizotinib, 1 ceritinib and 1 alectinib), 18 (19%) patients received upfront crizotinib as switch after 1–2 cycles of pemetrexed-platinum-based chemotherapy, 6 (6%) patients received crizotinib as maintenance after 4–6 cycles of pemetrexed-platinum-based chemotherapy, 26 (26.8%) patients received upfront chemotherapy, while ALK TKI could be used in subsequent lines in seven of these patients. Two (2%) patients received only supportive care due to poor PS precluding any systemic therapy. Thus, ALK inhibitors could be used in 74 (76.3%) patients with poor PS in this study.

### Progression‑free survival

**Figure 2.**
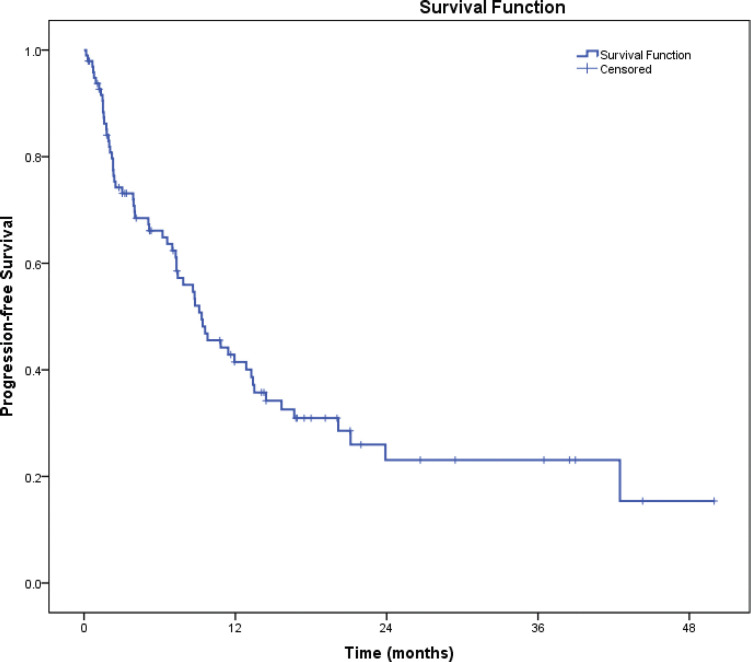
PFS.

The median follow-up duration was 22.4 months. On intention to treat analysis, median PFS on first-line therapy for overall population was 14.1 months (95% CI = 12.3–15.9). The median PFS for patients with PS 0–1 was 14.9 months (95% CI = 13.4–16.4) versus 9.3 months (95% CI = 6.6–12.0) in patients with poor PS (2–4) (HR = 1.38, 95% CI = 1.04–1.84, p=0.027, Figure 2).

### Overall survival

**Figure 3.**
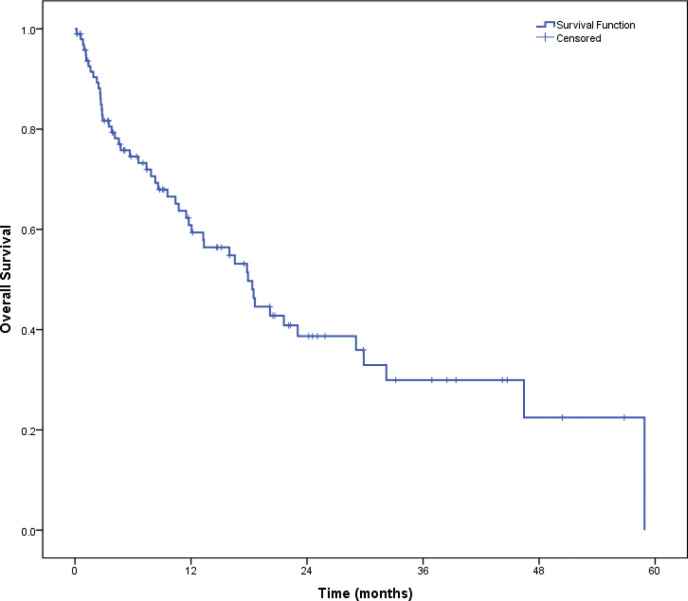
OS.

**Figure 4.**
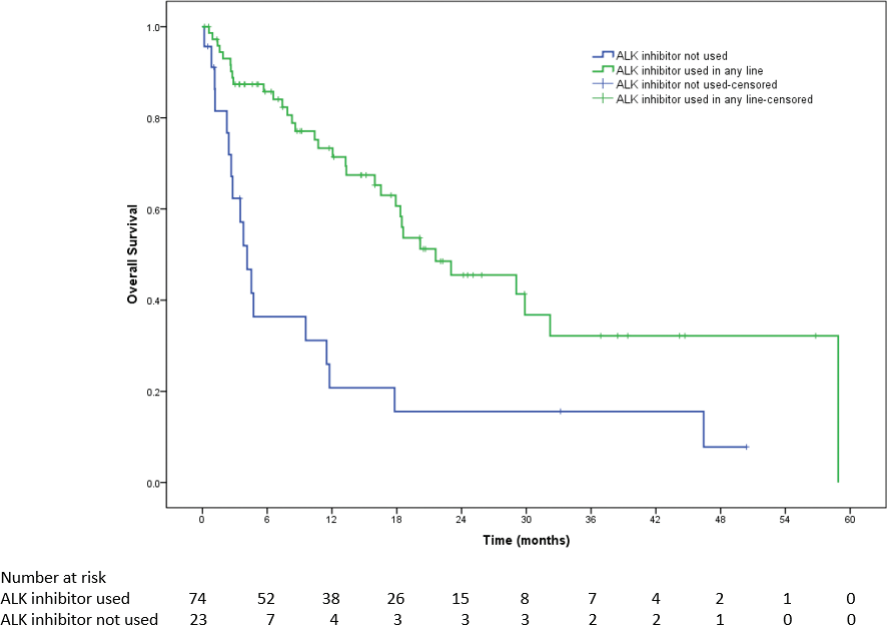
Kaplan–Meier survival curve showing OS in poor performance status patients who could receive ALK inhibitors in any line versus those who could not.

The median OS was 31.6 months (95% CI = 28.3–34.8). Median OS in patients with PS 0–1 was 33.5 months (95% CI = 28.6–38.4) versus 17.9 months (95% CI = 12.8–23.1) (HR = 1.89, 95% CI = 1.36–2.62, *p* < 0.001, Figure 3) in patients with poor PS. The median OS for patients who could receive ALK inhibitors in any line was 21.6 months (95% CI = 11.3–31.8) versus 4.1 months (95% CI = 2.7–5.6) for those who could not receive ALK inhibitors (HR = 2.9, 95% CI = 1.6–5.3, *p* < 0.001, Figure 4).

Univariate analysis of the various factors affecting OS found that ECOG PS 3–4 (versus PS 2), smokers and ALK inhibitors not received in first line led to significantly poorer outcomes. ECOG PS 3–4 was associated with HR = 1.79 (95% CI = 1.01–3.20, *p* = 0.047) as compared to PS 2; smokers had HR = 2.07 (95% CI = 1.0–4.3, *p* = 0.05) as compared to non-smokers, while the use of ALK inhibitors in first line was associated with HR = 0.49 (95% CI = 0.28–0.88, *p* = 0.014) as against not used in first line (Table 1). On multivariate analysis, the use of ALK inhibitors in the first line was the only significant factor with HR = 0.55 (95% CI = 0.31–0.99, *p* = 0.049).

### PS 2 versus PS 3–4

**Figure 5.**
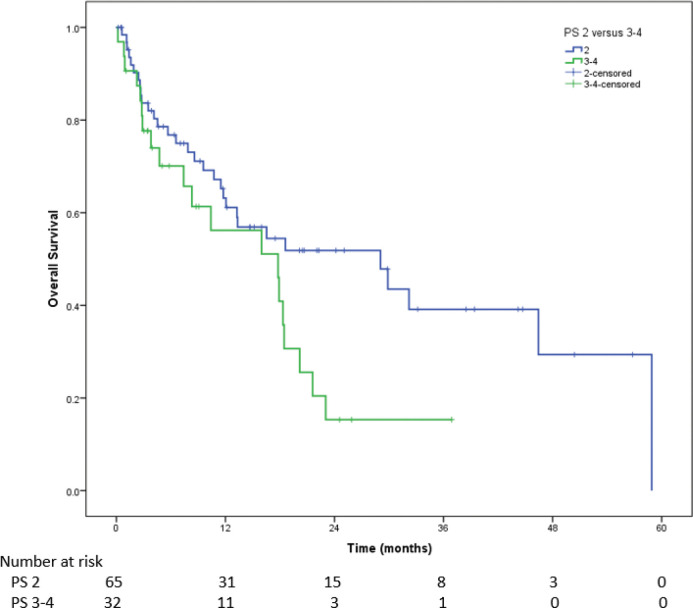
For PS 2, the median OS was 20.6 months (95% CI = 3.8–37.5) and for PS 3–4 it was 8.6 months (95% CI = 6.1–11.4) (*p* = 0.010)

Among patients with PS 2, the median PFS on first-line therapy was 11.4 months (95% CI = 7.2–15.6) as against 7.4 months (95% CI = 3.3–11.6) for patients with PS 3–4 (HR = 1.71, 95% CI = 1.02–2.88, p=0.043). Among patients with PS 2, median OS was 20.6 month (95% CI = 3.8–37.5) versus 8.6 month (95% CI = 6.1–11.4) in patients with PS 3 or 4 (*p* = 0.010) (Figure 5). For patients with PS 3–4, the median OS for patients who could receive ALK inhibitors (n=24) was 18.3 months (95% CI = 17.3–19.3), while it was 2.8 months (95% CI = 1.4–4.1) for those could not receive ALK inhibitors (HR = 6.8, 95% CI = 2.3–20.0, *p* < 0.001). The Wilcoxon signed-rank test showed that first-line therapy elicited a statistically significant change in PS (*Z* = −7.389, *p* < 0.001). Indeed, the median PS improved from 2 at baseline to 1 post-first-line therapy.

### TKI versus chemotherapy

The median PFS was 20 months in patients who received upfront TKI or as switch after platinum-based chemotherapy versus 8.8 months who received upfront chemotherapy. The median OS was 29.8 months (95% CI = 10.4–49.4) in patients who received upfront TKI or as switch after initial chemotherapy versus 11.7 months (95% CI = 1.8–21.7) who received upfront chemotherapy (HR = 0.49, 95% CI = 0.28–0.88, *p* = 0.016).

### Toxicities

Out of 97 patients, 70 (72.2%) patients received crizotinib at some point during their treatment. Transaminitis was the most common adverse effect with grade ½ in 42 (60%) patients and grade ¾ in 3 (4.3%) patients (Table 2). Anaemia was the next most common adverse effect with grade ½ in 32 (42.7%) patients. Peripheral oedema developed in 22 (31.4%) patients, QTc prolongation in 16 (22.8%) patients, rise in creatinine in 7 (10%) patients, renal cysts formation in 2 (2.8%) patients and visual hallucinations occurred in 4 (5.7%) patients. Dose reduction was required in 2 (2.8%) patients receiving crizotinib due to recurrent grade 3 transaminitis. No patient required withdrawal of ALK inhibitors due to toxicities.

## Discussion

The prognosis of patients with ALK-rearranged NSCLC has improved in the last decade, and the median survival time is over 5 years in advanced stages with the use of third-generation ALK inhibitors [11]. Although chemotherapy is not recommended in patients with poor PS, it is feasible to use ALK inhibitors even in these patients. In this study, we report median OS of 21.4 months in patients with poor PS who could receive ALK inhibitors in any line. This is clearly much higher than any previously described results of palliative chemotherapy or supportive care alone [12, 13]. In a phase 2 study (*n* = 18) by Iwama *et al* [14], alectinib showed an ORR of 72% and median PFS of 10.2 months in patients with PS 2–4. They found no significant difference in PS 2 versus PS 3–4. This is in contrast to our study in which there are significant differences in the two groups in terms of both PFS and OS. This can be explained by real-world data with intention-to-treat analysis in our study where all patients with poor PS are included, while in the study by Iwama *et al* [14], only patients with poor PS who received alectinib were included. The main concern with chemotherapy in poor PS patients remains the risk of increased toxicities [1]. However, in our study, we did not find any significant increase in toxicities with ALK inhibitors, despite the PS being 2–4. This is in agreement with the results of the use of alectinib in similar patients. Considering the available data, the National Comprehensive Cancer Network (NCCN) guidelines recommend the use of ALK inhibitors as first-line or subsequent therapy in patients with PS 0–4 [15].

The use of ALK inhibitors in first line came as the only significant factor in multivariate analysis for OS. The data of use in first line included patients who were initially started on chemotherapy but later shifted to ALK inhibitors after few cycles. This highlights the importance of using ALK inhibitors in first line instead of reserving them for second line or beyond. This is line with the data of PROFILE 1014 published in 2014, showing PFS at 18 months to be 31% (95% CI = 23–39) in the crizotinib arm and 5% (95% CI = 2–10) in patients who received chemotherapy in the first line [16]. This difference in PFS translated into OS benefit as well, with final analysis published in 2018 showing an improvement in OS favouring crizotinib (HR = 0.34; 95% bootstrap CI = 0.08–0.72) over chemotherapy after adjusting for cross-over [17].

Also, our study showed that the majority of poor PS patients had extensive metastatic disease at presentation which may be related to the EML4-ALK variant 3 [18]. This variant is associated with poor survival outcome, which may explain shorter OS and PFS in our poor PS population as opposed to patients with preserved PS at presentation. A limitation to our data is that the method of ALK testing was predominantly by IHC method and few by FISH method, hence it is difficult to make any final conclusion.

There are few important limitations of the study, which include the obvious drawbacks of a retrospective audit limiting the recording of toxicities, besides the treatment being heterogeneous. However, this study adds important real-world data in the treatment of ALK-rearranged NSCLC and gives confidence to the clinicians in prescribing ALK inhibitors to patients with poor PS at baseline.

## Conclusion

ALK-rearranged NSCLC patients with poor PS derived significant benefits with ALK inhibitors. The outcomes were significantly poorer as compared to patients with PS 0–1; the subgroup of patients with PS 2 had better outcomes as compared to patients with PS 3–4.

## Ethical statement

The study was conducted in accordance with the Declaration of Helsinki and the International Conference on Harmonisation Guidelines for Good Clinical Practice.

## Funding declaration

No funding was received for this study.

## Conflicts of Interest

The authors declare that they have no competing interests.

## Figures and Tables

**Table 1.** Univariate and multivariate analyses for OS by Cox regression method.

Factors	Number (*n*)	Univariate HR (95% CI)	*p*-value	Multivariate HR (95% CI)	*p*-value
**Age >60 years** No Yes	68 29	Ref 1.53(0.80–2.94)	0.195	**-**	**-**
**Gender** Female Male	43 54	Ref 0.97 (0.73–1.28)	0.836	.-	**-**
**Smoker** No Yes	85 12	Ref 2.07 (1.00–4.31)	**0.050**	Ref 1.73 (0.82–3.65)	0.150
**ECOG PS** 2 3–4	65 32	1.79 (1.01–3.20)	**0.047**	Ref 1.72 (0.96–3.06)	0.067
**Comorbidities** No Yes	60 37	Ref 1.51 (0.82–2.77)	0.187	**-**	**-**
**Stage ** III IV	6 91	Ref 2.52 (0.61–10.4)	0.201		
**Liver metastasis** No Yes	83 14	Ref 1.02 (0.48–2.20)	0.955	**-**	**-**
**Brain metastasis** No Yes	72 25	Ref 1.05 (0.56–1.99)	0.863	**-**	**-**
**ALK inhibitor used in first line** No Yes	44 53	Ref 0.49 (0.28–0.88)	**0.016**	Ref 0.55 (0.31–0.99)	**0.049**

Smoking and ECOG PS, on univariate analysis shown significant association with survival outcome, but not on multivariate analysis there was no statistical association. ALK inhibitor therapy has shown statistically significant association on both univariate and multivariate analysis

**Table 2.** Adverse effects of crizotinib in ALK-rearranged non-small cell lung cancer patients with baseline poor performance status (numbers in brackets indicate percentages).

Adverse effect	Patients receiving crizotinib (n=70)	
	Grade 1/2	Grade 3
Anaemia	32 (42.7)	1 (1.4)
Neutropenia	1 (1.4)	9 (12.8)
Thrombocytopenia	4 (5.7)	0
Transaminitis	42 (60.0)	3 (4.3)
Fatigue	18 (25.7)	3 (4.3)
Vomiting	21 (30.0)	2 (2.8)
QTc prolongation	14 (20.0)	2 (2.8)
Peripheral oedema	22 (31.4)	0
Rash	4 (5.7)	0
